# Tracking the kinetics of intrahepatic immune responses by repeated fine needle aspiration of the liver

**DOI:** 10.1016/j.jim.2015.04.011

**Published:** 2015-09

**Authors:** Tom Pembroke, Awen Gallimore, Andrew Godkin

**Affiliations:** Institute of Infection and Immunity, School of Medicine, Cardiff University, UK

**Keywords:** Natural killer cells, Fine needle aspiration, Intrahepatic lymphocytes

## Abstract

Liver disease is an increasing global health burden. The final sequalae of cirrhosis, liver failure and hepatocellular carcinoma are often the result of inflammation driven by intrahepatic lymphocytes. Accurate assessment of organ-specific diseases ideally employs tissue sampling though this is rarely performed. Here we report our experiences of utilising repeated fine needle aspirations (FNAs) to assess liver-derived leukocytes. In 88 patient samples, we obtained a mean of 36,959 lymphocytes from each FNA-derived biopsy (SD 22,319 cells, range 5034–91,242 cells) measured by flow cytometry. This quick technique required minimal analgesia compared to liver biopsy (p = 0.03); was well tolerated and safe, and hence repeated sampling up to 3 times within a week was feasible. We detail the technique to rapidly derive a single cell suspension suitable for multiparameter flow cytometry analysis. Finally we illustrate the importance of organ-derived sampling by showing that natural killer (NK) cells from FNA samples have a markedly altered phenotype compared to those assessed in peripheral blood. In combination these data validate FNA as a powerful and well-tolerated method of sampling intrahepatic lymphocytes to study the immunology of acute and chronic liver diseases.

## Highlights

• Hepatic fine needle aspiration (FNA) is a safe, well-tolerated technique.
• FNA allows repeated sampling of the intrahepatic compartment from the same individual.
• A mean number of 37,000 lymphocytes are aspirated from the liver.
• Analgesic requirements are minimal after undergoing FNA compared to liver biopsy.

## 1 Introduction

Inflammatory liver diseases, including chronic viral hepatitis, autoimmune hepatitis and steatohepatitis, represent a major global health burden. Lymphocytes are often stated to be the drivers of these clinical disorders, but direct evidence is often lacking. It is well recognised that, although easy to sample, peripheral blood lymphocytes may not reflect the function and phenotype of cells within the liver. Sampling cells from within the liver has remained problematic. Liver biopsy, using 16–18 gauge sheathed (Tru Cut) needles or suction (Menghini) needles, remains the clinical gold standard to assess liver fibrosis and inflammation and can provide valuable diagnostic information. However, the invasive nature of the liver biopsy, coupled with significant morbidity (and very rarely mortality) has driven interest in development of biochemical and non-invasive markers (i.e. liver elastography) to assess chronic liver disease (Castera, 2012). Percutaneous liver biopsy has a mortality rate of 1 in 10,000 and the most common complication is abdominal or right shoulder pain in up to 25% of patients (Bravo et al., 2001). Whilst the parenchyma of the liver has a relatively paucity of nerve fibres the liver capsule is well innervated and together with the skin requires infiltration with local anaesthetic; in spite of this some patients require additional analgesia. Other rare (< 1%) risks associated with liver biopsy include bleeding, pneumothorax, perforation and peritonitis (Bravo et al., 2001).

To gain a sufficient number of lymphocytes for analysis, it has been necessary to obtain a separate biopsy core designated for research purposes. The risk of complications from liver biopsy obviously increases with the number of passes of the needle into the liver. This separate core requires physical and enzymatic degradation to isolate lymphocytes. The clinical risks associated with liver biopsy prevent the use of this technique to monitor lymphocytes at frequent intervals within the same individual (Ahlenstiel et al., 2011). During interferon-based treatment of viral hepatitis, we and others have found that the largest reductions in viral load take place during the first week, thus it would be useful to closely monitor intrahepatic changes during this period of marked perturbation (Davis et al., 2003, Pembroke et al., 2012).

These limitations to the use of standard liver biopsy techniques led us to explore the use of Fine Needle Aspiration (FNA) to obtain samples. FNA needles rely on aspiration of a clinical sample via a small-bore needle (usually 23 gauge). This is a clinical technique that provides single cell cytology samples and as such is unable to provide histological information. However, it is reliable, relatively safe and often provides the required diagnostic information. FNA has been used to obtain lymphocytes from organs to monitor disease (Oliveira et al., 1997, Claassen et al., 2011).

Aspiration of tissue from within a solid organ, including from within the liver capsule, will inevitably contain a mixture of blood cells and cellular debris from the stroma. A hurdle to the use of FNA as a technique to sample intrahepatic lymphocytes is the need to remove sampling debris to enable effective labelling of lymphocytes and analysis by flow cytometry. In this report we discuss our experiences of performing 88 liver FNAs to sample intrahepatic lymphocytes, including repeat FNA sampling on a subset of 9 patients. We discuss the practicality of this approach with respect to patients' tolerance, the procedure itself and sample processing. We also highlight comparative datasets obtained from intrahepatic and blood lymphocytes derived from the same individuals.

## 2 Methods

### 2.1 Ethics statement

South East Wales Local Research Ethics Committee reviewed this project (10/WSE02/45). Patients who participated were given a study information sheet and provided informed written consent.

### 2.2 Study subjects

Fifty-three consecutive patients attending the hepatology department for outpatient liver biopsy or IFNα treatment of HCV were recruited. Liver biopsy was performed on 49 individuals as part of their routine workup whereas FNA alone was conducted on 4. Nine patients undergoing IFNα treatment were sampled by FNA a combined total of 35 times at days 1, 3, 7, and 14 & months 1, 3 and 6 following the initiation of treatment (Table 1).

FNA samples were taken in a day case treatment room in the University Hospital of Wales. Prior to the FNA, all patients underwent a recent ultrasound scan and α-fetoprotein (to exclude abnormal anatomy and intrahepatic lesions) and routine clinical bloods (full blood count, liver biochemistry, coagulation screen) were assessed. Patients were considered eligible for the procedure if platelets were > 60, 000/μl and prothrombin time < 14.5 s in keeping with local liver biopsy guidelines. Patients were seen on an outpatient basis with a view to discharge following the procedure. Clinicians experienced in biopsying the liver undertook this procedure. Written informed consent was obtained—the risks we described relating to the FNA procedure are as follows:

• Common: approximately 10–25% of procedures:
  ○ Bruising and soreness
• Serious complications: < than 1 in every 10,000 procedures:
  ○ Bleeding requiring blood transfusion
  ○ Damage to organs surrounding the liver—including the lung, bowel and gallbladder
  ○ Peritonitis
  ○ Pneumothorax

Separate written consent for liver biopsy was obtained in the usual manner for those undergoing both procedures.

### 2.3 Materials for Fine Needle Aspiration

1. Roswell Park Memorial Institute (RPMI, Gibco, Paisley UK) media supplemented with 10% foetal calf serum, 250 μl heparin, 100 U/ml penicillin, 100 μg/ml streptomycin, 2 mM L-glutamine and 2 mM sodium pyruvate (R-10), stored on ice.
2. Sterile dressing pack (Rocialle, Mountain Ash UK) including gloves, gauze swabs, gallipot and sterile paper field.
3. 1% tincture of iodine solution (Videne).
4. 5 and 10 ml syringes.
5. Orange (25 gauge), Green (21 gauge) and White (19 gauge) hypodermic needles (BD Oxford UK).
6. 10 ml 2% lignocaine (Braun, Melsugen Germany).
7. 22 gauge spinal needle Quinke type point with an internal trocar (BD, Oxford UK).
8. Sterile dressing for puncture site (Premier, Enfield UK).

### 2.4 Fine Needle Aspiration technique

We have developed the following protocol for FNA sampling of intrahepatic lymphocytes. Standard aseptic clinical procedure is observed using a plastic disposable apron, sterile gloves and sterile disposable towels.

1. Obtain a heparinised blood sample for density gradient isolation of lymphocytes as previously described (Gallagher et al., 2009).
2. Place the patient in the supine position with the right hand behind the head. Percuss the lower edge of the liver in the right mid-axillary line and mark suitable site 1–2 intercostal spaces above the lower edge; this is usually 2 intercostal spaces above the costal margin. Confirm that the marked position for FNA sampling over the liver is dull on percussion in full expiration.
3. Clean the skin with iodine solution.
4. Draw up 5 ml 2% lignocaine into a 5 ml syringe; inject 1–2 ml into the dermis with a 25-gauge orange needle. Allow the lignocaine to take effect (30–60 s), then replace this needle with a 23-gauge green needle and continue to infiltrate 2% lignocaine through the intercostal muscles to the hepatic capsule. Occasionally a longer 21 gauge white needle is required for larger patients.
5. Insert the 22-gauge spinal needle with internal trocar in situ along the anaesthetised tract to the edge of liver capsule. Instruct the patient to inhale, then exhale fully and hold their breath in full expiration. The needle can then be inserted 2–3 cm into the liver parenchyma from the capsule.
6. Remove the internal trocar and attach a 10 ml syringe filled with ice-cold R-10 and aspirate using gentle negative syringe pressure as the needle is withdrawn 1–2 cm but remaining within the liver parenchyma.
7. Remove the needle and draw fresh media into the syringe to ensure that the entire aspirate passes into the syringe.
8. Detach the syringe and replace the trocar and insert the needle through the same anaesthetised tissue but orientated in a slightly different direction when passing into the liver. Repeat the aspiration as in (6) and (7).
9. The contents of the syringe are promptly passed into a 50 ml Falcon tube on ice containing a further 20 ml R-10 and then transported to the lab for immediate staining as described in below.
10. Apply a simple plaster over aspiration site. Dress the wound site. The patient can be discharged in < 30 min if there are no adverse effects.

Patients can resume normal daily activities. We advised the patient to keep the wound site clean and dry, and avoid heavy lifting, for 24 h.

### 2.5 Preparation of samples for flow cytometry

Peripheral blood mononuclear cells (PBMC) were isolated using a Ficoll density gradient. Various techniques were employed to separate intrahepatic lymphocytes from debris associated with the FNA sampling.

#### 2.5.1 Method 1

Initially samples were passed through a 70 μm cell strainer (Fischer Scientific, Loughborough UK) and centrifuged at 1600 RPM for 5 min. The pellet was resuspended in 5 ml of R-10 and layered over Ficoll and centrifuged for 2000 RPM for 20 min. Although an interface was not evident the solution above the Ficoll layer was harvested and stained as described below.

#### 2.5.2 Method 2

Samples were centrifuged at 1600 RPM for 5 min and resuspended in cold phosphate buffered saline (PBS). This step was repeated and cells were subsequently plated for fluorochrome labelled monoclonal antibody staining. Cell surface markers were stained. Debris was removed during 0.5% saponin permeabilisation (ebioscience, Hatfield UK) and samples were washed 3 times in permeabilisation buffer (ebioscience, Hatfield UK). Intracellular cytokines were then stained using labelled antibodies.

### 2.6 Immunophenotyping and intracellular staining

Both intrahepatic lymphocytes and PBMC were washed twice in PBS and stained with Aqua live/dead stain (Invitrogen, Paisley, UK) and fluorochrome labelled monoclonal antibodies specific for CD3-APCH7, (BD Biosciences, Oxford) CD16-FITC, (eBioscience, Hatfield, UK) and CD56-PerCP.Cy5 (Biolegend, Cambridge, UK). Cells were then permeabilised and fixed with Fix/Perm solution (eBioscience, Hatfield, UK) according to the manufacturer's instructions, washed and stained with antibodies specific for granzyme B-APC (Invitrogen, Paisley UK) and IFNγ-e450 (eBioscience, Hatfield, UK). Cells were washed and fixed with 2% paraformaldehyde solution and analysed by flow cytometry as described above.

### 2.7 Statistical analysis

Comparisons of lymphocyte numbers and phenotypic markers were made using either the Student *t*-test (assuming normal distribution of data) or the Mann–Whitney *U* test (for data without normal distribution). χ^2^ test was used to compare analgesia requirements between the FNA and biopsy groups. The software employed was Excel 2011 and GraphPad Prism 5.0.

## 3 Results and discussion

### 3.1 Results

#### 3.1.1 Patient outcome and side effects

**Fig. 1:**
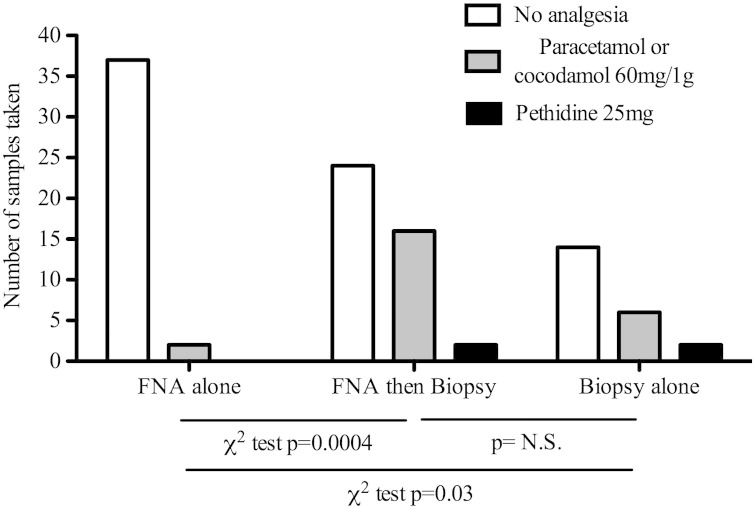
Post procedure analgesia. Analgesia was offered to patients at regular intervals following FNA and liver biopsy procedures. 2 patients requested simple analgesia after FNA alone; 16 patients who underwent FNA followed by biopsy requested simple analgesia and 2 required pethidine p = 0.0004 χ^2^ test. Of 22 patients who had biopsy alone 6 required cocodamol and 2 required pethidine (v FNA alone p = 0.025 χ^2^ test); there was no significant difference in analgesia requirement in biopsy alone v biopsy and FNA (p = ns χ^2^ test).

The FNA sampling technique described here was applied to a cohort of 53 patients. Nine patients had FNA samples taken at multiple time points (Table 1) resulting in a total of 81 intrahepatic samples obtained for lymphocyte analysis. The procedure is well tolerated. Following the 39 FNAs alone (i.e. did not proceed to Tru-Cut liver biopsy) 1 patient required 1 g of paracetamol and 1 patient received 30 mg of codeine and 500 mg of paracetamol. When patients proceeded to liver biopsies following FNA there was a marked increase in the need for analgesia compared to FNA alone, with 16 patients requiring cocodamol and 2 patients required intramuscular pethidine (χ^2^ p = 0.0004, Fig. 1). Twenty-two consecutive patients who had biopsy alone were reviewed. The analgesia requirements of individuals who had FNA and biopsy were not increased compared to those who had biopsy alone. Individuals who had FNA alone had a significantly lower analgesia requirement than those who had biopsy alone (χ^2^ p = 0.03 Fig. 1). Of note a significant proportion of patients (9/12) who commenced treatment with IFNα agreed to repeated FNA sampling with up to three samples taken during the first week. There were no other complications associated with the FNA sampling or liver biopsies in this cohort.

#### 3.1.2 Phenotype of lymphocytes purified after FNA

**Fig. 2:**
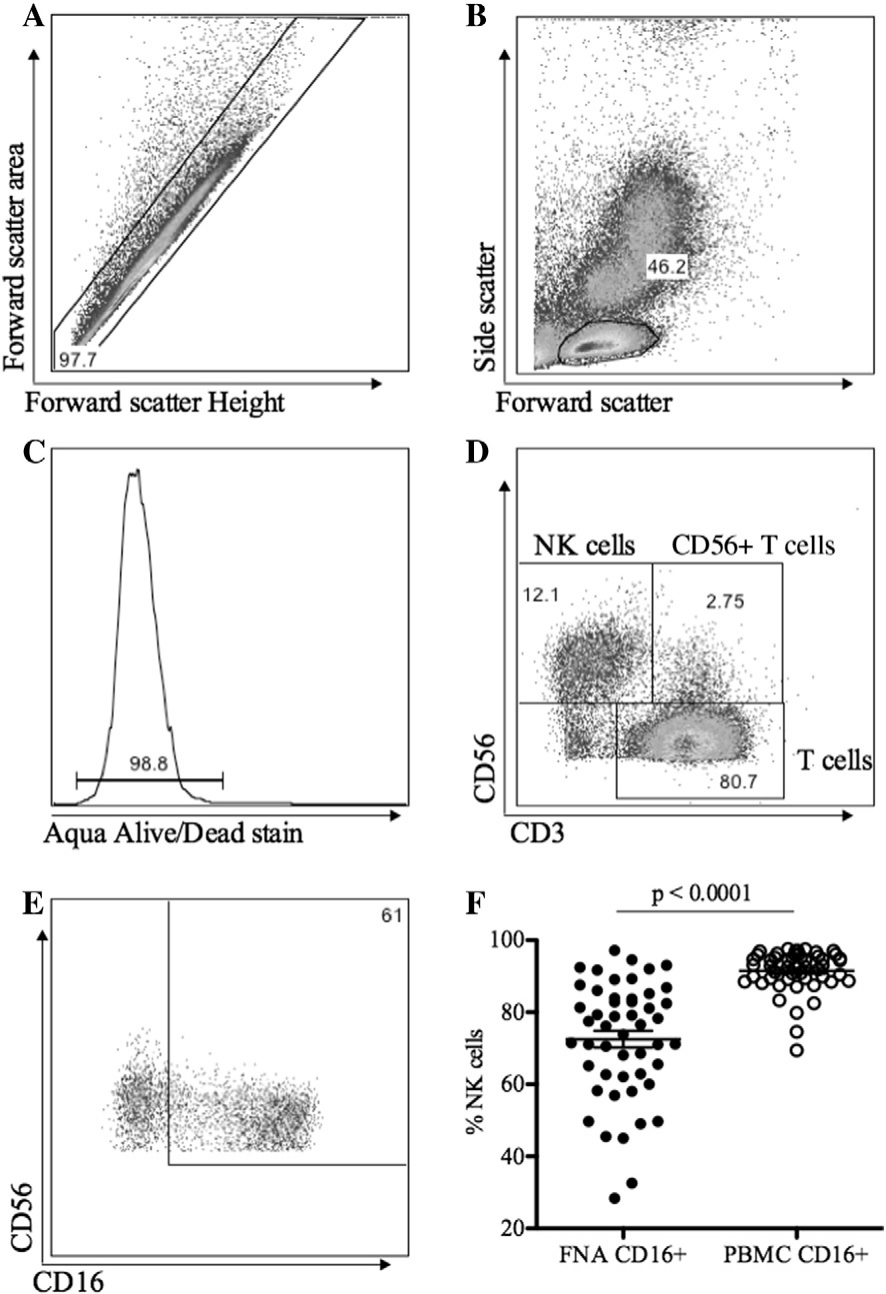
Lymphocyte gating strategy. Intrahepatic lymphocytes were stained with fluorochrome labelled monoclonal antibodies and analysed by flow cytometry. A) Single cells were identified by forward scatter height and area. B) Lymphocytes were gate based on forward and side scatter. C) Aqua staining to exclude dead cells. D) CD56^+^ CD3^−^ NK cells, CD56^+^ CD3^+^ NKT cells and CD3^+^ T lymphocytes. E & F) intrahepatic NK cells had markedly reduced CD16 expression compared to the peripheral blood.

Two methods to isolate intrahepatic lymphocytes from the aspirate were tested. As described in Materials and methods, the first method required layering aspirate upon Ficoll and isolating lymphocytes using a density gradient. The second method utilised saponin permeabilisation and repeated washing to clear cell debris. In both methods, cells were stained with monoclonal antibodies and phenotype and function were assessed using flow cytometry as follows: Single cells were selected using forward scatter height and forward scatter area gating. Lymphocytes were then gated based on forward and side scatter and dead cells were excluded with aqua live dead stain. Lymphocyte subsets (NK, NKT and T lymphocytes) were gated based upon expression of CD56 and CD3 (Fig. 2A–E). Compared to NK cells in matched PBMC samples, the phenotype of intrahepatic NK cells retrieved by FNA was markedly altered. Whilst the proportion of CD56^Dim^ NK cells was unaltered, the proportion of CD16^+^ NK cells was significantly lower (p < 0.0001, Fig. 2F) indicating that distinct NK cell populations were obtained (Pembroke et al., 2014).

**Fig. 3:**
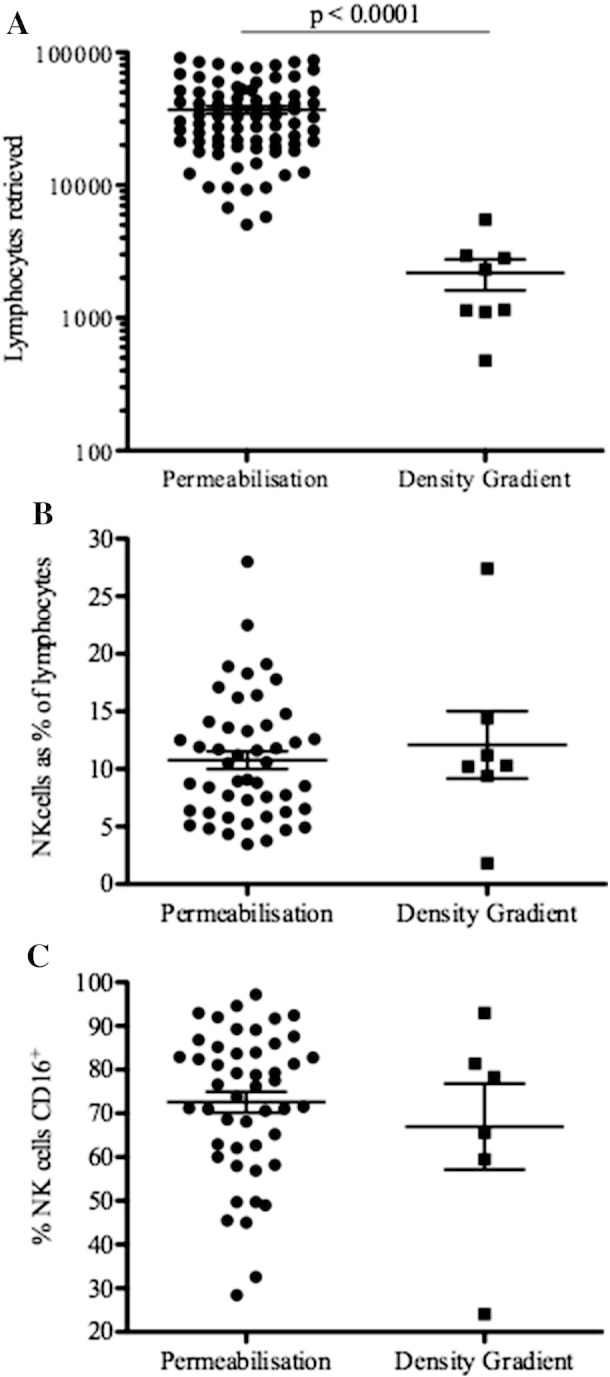
Intrahepatic lymphocyte isolation by permeabilisation and density gradient techniques. Lymphocytes isolated from FNA aspirates were prepared by cell permeabilisation and washing, which broke up cell debris and by Ficoll density gradient. A) Total number of lymphocytes retrieved. B) The proportion of NK cells of total lymphocytes retrieved. C) NK cell CD16 expression using both techniques. Mean and SEM shown.

The number of lymphocytes retrieved by the saponin permeabilisation and density gradient methods were compared. The number of intrahepatic lymphocytes retrieved was significantly higher using permeabilisation compared to a density gradient (mean lymphocytes = 36,959 v 2183 respectively p < 0.0001, Fig. 3A). The proportion of NK cells was similar using both methods (10.8% v 12.1% p = 0.7, Fig. 3B). Whilst it is difficult to accurately assess the phenotype of such small numbers of cells retrieved using density gradient separation, proportions of CD16^+^ NK cells were similar with both methods (72.5% v 67.0% p = 0.46, Fig. 3C).

### 3.2 Discussion

In this report we describe in detail a FNA technique to sample intrahepatic lymphocytes. It is important to note that these FNA samples are taken from the intracapsular space. As the liver is an extremely vascular organ it is likely that the needle may have passed through small sinusoids. If samples were heavily blood stained then they were discarded and the needle passed into fresh liver, this occurred on less than 5 passes of the spinal needles during the study period. The marked alteration in NK cell phenotype confirms that we were sampling a truly separate population to the peripheral blood, as we have described previously (Pembroke et al., 2014).

These protocols can be utilised to sample and study lymphocytes from the intrahepatic compartment providing novel insights into inflammatory liver diseases. The number of lymphocytes retrieved allows a phenotypic and functional analysis by flow cytometry. Other potential applications include gene expression analysis, protein arrays, and functional T-cell assay e.g. ELISpots. This procedure is well tolerated and acceptable to patients allowing collection of multiple samples during a week, thereby providing a unique insight into the kinetics of intrahepatic responses (Pembroke et al., 2014).

## 4 Conclusion

In conclusion, the protocol presented in this paper describes a simple approach for isolation of intrahepatic lymphocytes. These cells can be easily prepared for analysis by flow cytometry or other techniques. The application of this technique can provide samples for a broad range of immunological assays. We anticipate that this protocol will be helpful in studies of a range of inflammatory liver disorders.

## Figures and Tables

**Table 1:** FNA donor characteristics.

Characteristics	Chronic liver disease	HCV
Number	29	24
Mean age (range)	50.6 (29–69)	49 (27–65)
Male:female	16:13	17:7
Median viral load (range)	–	1.3 × 10^6^ (1.9 × 10^3^–3 × 10^7^)
Median NI score (range)	1 (0–6)	5 (2–8)
Median fibrosis score (range)	2 (0–6)	2 (0–2)
HCV genotype		
1 3	– –	16 8

## References

[bb0005] Ahlenstiel G., Edlich B., Hogdal L.J., Rotman Y., Noureddin M., Feld J.J., Holz L.E., Titerence R.H., Liang T.J., Rehermann B. (2011). Early changes in natural killer cell function indicate virologic response to interferon therapy for hepatitis C. Gastroenterology.

[bb0010] Bravo A.A., Sheth S.G., Chopra S. (2001). Liver biopsy. N. Engl. J. Med..

[bb0015] Castera L. (2012). Noninvasive methods to assess liver disease in patients with hepatitis B or C. Gastroenterology.

[bb0020] Claassen M.A., de Knegt R.J., Janssen H.L., Boonstra A. (2011). Retention of CD4 + CD25 + FoxP3 + regulatory T cells in the liver after therapy-induced hepatitis C virus eradication in humans. J. Virol..

[bb0025] Davis G.L., Wong J.B., McHutchison J.G., Manns M.P., Harvey J., Albrecht J. (2003). Early virologic response to treatment with peginterferon alfa-2b plus ribavirin in patients with chronic hepatitis C. Hepatology.

[bb0030] Gallagher K.M., Lauder S., Rees I.W., Gallimore A.M., Godkin A.J. (2009). Type I interferon (IFN alpha) acts directly on human memory CD4 + T cells altering their response to antigen. J. Immunol..

[bb0035] Oliveira J.G., Ramos J.P., Xavier P., Magalhaes M.C., Mendes A.A., Guerra L.E. (1997). Analysis of fine-needle aspiration biopsies by flow cytometry in kidney transplant patients. Transplantation.

[bb0045] Pembroke T., Rees I., Gallagher K., Jones E., Mizen P., Navruzov T., Freedman A., Fielding C., Humphreys I.R., Wang E.C., Gallimore A.M., Godkin A. (2012). Rapid early innate control of hepatitis C virus during IFN-alpha treatment compromises adaptive CD4 + T-cell immunity. Eur. J. Immunol..

[bb0040] Pembroke T., Christian A., Jones E., Hills R.K., Wang E.C.Y., Gallimore A.M., Godkin A. (2014). The paradox of NKp46+ natural killer cells: drivers of severe hepatitis C virus-induced pathology but in-vivo resistance to interferon alpha treatment. Gut.

